# Phenotypic clustering: a novel method for microglial morphology analysis

**DOI:** 10.1186/s12974-016-0614-7

**Published:** 2016-06-17

**Authors:** Franck Verdonk, Pascal Roux, Patricia Flamant, Laurence Fiette, Fernando A. Bozza, Sébastien Simard, Marc Lemaire, Benoit Plaud, Spencer L. Shorte, Tarek Sharshar, Fabrice Chrétien, Anne Danckaert

**Affiliations:** Human Histopathology and Animal Models Unit, Infection and Epidemiology Department, Institut Pasteur, Paris, France; Imagopole - CITech, Institut Pasteur, Paris, France; ICU, Instituto de Pesquisa Clínica Evandro Chagas, Fundação Oswaldo Cruz, Rio de Janeiro, Brazil; Air Liquide Santé International, World Business Line Healthcare, Medical R&D, Paris-Saclay Research Center, 1 chemin de la Porte des Loges, Jouy-en-Josas, France; Department of Anaesthesiology and Surgical Intensive Care, Saint-Louis University Hospital of Paris, Paris, France; Department of Intensive Care, Raymond Poincare University Hospital, Garches, France; Laboratoire hospitalo-universitaire de Neuropathologie, Centre Hospitalier Sainte Anne, Paris, France; Versailles Saint Quentin University, Versailles, France; Paris Diderot University, Paris, France; Paris Descartes University, Sorbonne Paris Cité, Paris, France; TRIGGERSEP, F-CRIN Network, Toulouse, France

**Keywords:** Microglial cell morphology, Neuroinflammation, Automated high-content analysis, Clustering, Sub-population behaviour, Complexity index

## Abstract

**Background:**

Microglial cells are tissue-resident macrophages of the central nervous system. They are extremely dynamic, sensitive to their microenvironment and present a characteristic complex and heterogeneous morphology and distribution within the brain tissue. Many experimental clues highlight a strong link between their morphology and their function in response to aggression. However, due to their complex “dendritic-like” aspect that constitutes the major pool of murine microglial cells and their dense network, precise and powerful morphological studies are not easy to realize and complicate correlation with molecular or clinical parameters.

**Methods:**

Using the knock-in mouse model CX3CR1^GFP/+^, we developed a 3D automated confocal tissue imaging system coupled with morphological modelling of many thousands of microglial cells revealing precise and quantitative assessment of major cell features: cell density, cell body area, cytoplasm area and number of primary, secondary and tertiary processes. We determined two morphological criteria that are the complexity index (CI) and the covered environment area (CEA) allowing an innovative approach lying in (i) an accurate and objective study of morphological changes in healthy or pathological condition, (ii) an in situ mapping of the microglial distribution in different neuroanatomical regions and (iii) a study of the clustering of numerous cells, allowing us to discriminate different sub-populations.

**Results:**

Our results on more than 20,000 cells by condition confirm at baseline a regional heterogeneity of the microglial distribution and phenotype that persists after induction of neuroinflammation by systemic injection of lipopolysaccharide (LPS). Using clustering analysis, we highlight that, at resting state, microglial cells are distributed in four microglial sub-populations defined by their CI and CEA with a regional pattern and a specific behaviour after challenge.

**Conclusions:**

Our results counteract the classical view of a homogenous regional resting state of the microglial cells within the brain. Microglial cells are distributed in different defined sub-populations that present specific behaviour after pathological challenge, allowing postulating for a cellular and functional specialization. Moreover, this new experimental approach will provide a support not only to neuropathological diagnosis but also to study microglial function in various disease models while reducing the number of animals needed to approach the international ethical statements.

**Electronic supplementary material:**

The online version of this article (doi:10.1186/s12974-016-0614-7) contains supplementary material, which is available to authorized users.

## Background

Microglial cells are parenchymal tissue macrophages that account for 10 % of brain cells [1] involved in brain immune surveillance and homeostasis. In healthy conditions, they are involved in synaptic development and maintenance [2], neuronal survival [3] and phagocytosis [4] to maintain brain homeostasis. Microglia migrate to interact with other cell types (i.e. astrocyte and neurons) and produce a variety of factors required to induce neural progenitor differentiation [5] and neuronal apoptosis [6]. Their activation is the main component of the neuroinflammatory process, which can result either from direct brain insult or systemic inflammation leading to either neuroprotective [7, 8] or neurotoxic [9, 10] responses. Their activation is characterized by both immunological and morphological changes, including mainly a decrease in ramification up to the amoeboid form (i.e. large non-ramified cells). Hypotheses of an adaptation of their morphology to their specific function within the central nervous system (CNS) are formulated [11, 12] but neither could be actually substantiated due to two boundaries: (i) the difficulty-to-detect subtle morphological variations of microglia and also probably intermediate forms between the categories of activation that are historically described [13, 14] and (ii) the need to couple a very precise morphological single cell approach with a proteomic or transcriptomic study using specific techniques [15] taking into account the regionalization that is specific to the CNS. In fact, since the identification of microglial cells in 1932 by del Rio-Hortega [16], the assessment of microglial morphology allows characterizing their role or their level of activation [17, 18] but it remains challenging. The spectacular complexity of these cells, their morphological variability and their dense distribution within the tissue underlines the importance of a precise morphological characterization in a large number of cells distributed in the different functional CNS regions. The standard nowadays is the use of immunohistochemistry staining using CD45 or ionized calcium-binding adapter molecule 1 (Iba-1) localizing in the monocytic lineage and well expressed in microglia [19]. Those techniques, however, have some limitations. Indeed, since the expression of these markers depends on the intensity of microglial activation, immunostaining can be insufficient for accurately describing a “dendritic-like” or ramified phenotype [20, 21] that constitutes more than 90 % of the microglial cells in young mice [11]. Additionally, it required particular histological techniques such as paraffin embedding that may affect the precision of a morphological study. These limitations might be overcome by the use of transgenic mice, such as an Iba-1^GFP/+^ [22] or CX3CR1^GFP/+^ [23] mouse in which brain microglia express spontaneously green fluorescent protein (GFP) respectively under the control of the Iba-1 or the CX3CL1 (fractalkine) receptor locus. These transgenic models allow very precise visualization of the microglial ramifications requiring no immunostaining techniques. These models overcome the technical limits but ask the question of the quantification methods. Usually, quantification methods are based on semi-quantitative scoring and manual counting, making them time consuming, susceptible to inter- or intra-observer variability and imprecise. Furthermore, manual analyses are unable to assess either large numbers of cells or their network organization. Recently, innovative technical and/or mathematical methods have been developed allowing automated acquisition, fractal analysis [13] or segmentation of individual microglial cell shapes [24]. They have enabled to reliably assess the changes in microglial morphology albeit in limited numbers of cells (<70) [25–27]. Because of this limitation, the range of statistical analyses is also restricted while methods are available for analysing large numbers of cells and thus detecting subtle morphological phenotypes and changes therein. These methods are alternatives to conventional statistics and are able to exploit or highlight the major heterogeneity of cell populations in the same tissue, with an underlying organization that cannot be directly observed. Indeed, plasticity, reflected by slight morphological changes, is considered as a major functional property of microglial cells [28]. Using diverse criteria, it becomes now possible to discern precisely these sub-populations and structures by applying a clustering approach [29].

In efforts to develop a method allowing the assessment of the morphology of a large microglial population, and therefore for a better understanding of the microglial behaviour in different developmental, homeostatic and disease contexts, we herein propose an innovative strategy. Our approach is based on the automated acquisition of fluorescence and the measurement of morphological indexes in CX3CR1^GFP/+^ transgenic mice that allows discriminating microglial sub-populations based on a clustering analysis. To validate this method, we compared clustering analysis to parametric and non-parametric statistical approaches assessing their capacity to detect inter-regional variability and post-stimulation changes in microglial morphology. In conclusion, our approach is extremely efficient, reproducible and accurate.

## Methods

### Animals

Male and female C57BL/6 JRj mice purchased from JANVIER LABS and in-house CX3CR1^GFP/+^ mice aged from 9 to 11 weeks were used for these experiments. In the CX3CR1^GFP/+^ model, the CX3CL1 receptor gene, the CX3CR1, was knocked-in with a GFP reporter gene [23]. This gene is constitutively turned on in microglial cells and thus allows us to image them selectively using the GFP without any immunochemistry method. Mice were housed in cages in groups of seven, in a temperature- (22 ± 1.5 °C) and humidity-controlled environment, with a 12-h light/dark cycle. Mice were provided with food and water ad libitum according to international guidelines.

### Treatment conditions

Two experimental groups were considered. In the lipopolysaccharide (LPS) group (*n* = 6 by strain), the mice were injected intraperitoneally with 5 mg/kg of LPS from *Escherichia coli* serotype 055:B5 (Sigma-Aldrich) [30, 31] dissolved in 0.9 % saline. Twenty-four hours later, these mice were killed by cervical dislocation and the brain was collected. In parallel, mice belonging to the control group were not anaesthetized and were also killed by cervical dislocation before brain collection.

### Tissue preparation

After cervical dislocation, the brains were immediately removed and cut in a trans-sagittal plane in the inter-hemispheric fissure. Cerebral hemispheres were fixed during 24 h in 4 % buffered formalin (QPath, Labonord SAS, Templemars, France). Following fixation, tissue samples were sliced along a sagittal plane on a calibrated vibratome (VT1000 S, Leica, Germany) into 100-μm-thick free-floating slices. The most medial slices were used for analysis.

### Histological analysis and immunohistochemistry

Brain sections of the left hemisphere of C57BL/6 JRj mice were incubated with the rabbit antibody against Iba-1 (Wako Chemicals, Richmond, VA, 1:500) and revealed by the secondary antibody Dy488 (Jackson ImmunoResearch Laboratories, Baltimore, PA). A classical protocol was used: rehydratation, blocking with 20 % goat serum and 0.5 % Triton-X 100 for 2 h, incubation with primary antibody (Dako Diluent buffer, Glostrup, Denmark) overnight at 4 °C followed by incubation with secondary antibody 4 h at room temperature. The stained sections were mounted on slides and coverslipped

### Image acquisition and processing

Using a spinning disc confocal system (CellVoyager CV1000, Yokogawa, Japan) with a UPLSAPO 40×/NA 0.9 objective, sample areas were acquired as a square of 10 × 10 fields of view with a depth of 30 μm at 2-μm increments (16 focal depths) generating one volume in four regions of interest: striatum, frontal cortex, hippocampus and cerebellum. These regions were acquired sequentially allowing the coverage of approximately 3 mm^2^ of tissue per region. Each field corresponds to a matrix of 920 × 920 pixels; the pixel size in *X* and *Y* dimensions is 0.19 μm according to the objective. The 488-nm laser was used to excite GFP or detect Iba-1 and thus to image the microglial cells.

**Fig. 1:**
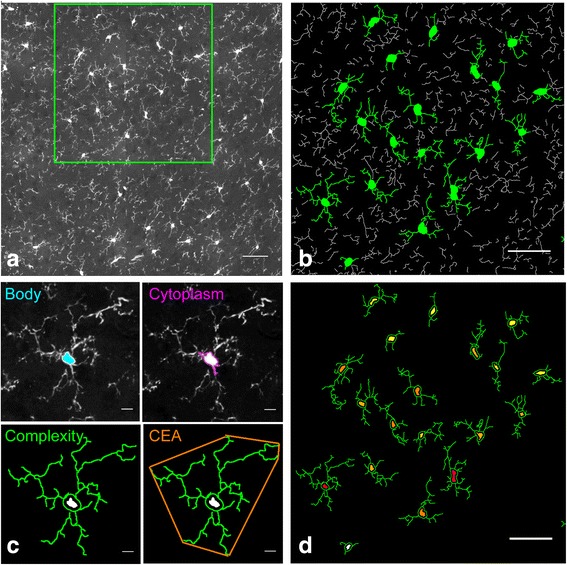
The characterization of microglial cells by morphological criteria. **a** Confocal images, representing a sub-part of the analysed image in the frontal cortex region after maximum intensity projection. The individual microglia based on GFP fluorescence appears in *white outline*. The scale bar equals 50 μm. **b** Ramification detection based on GFP fluorescence with Acapella^TM^ software. The segmented ramifications linked to an individual microglia are shown artificially in *green*, the unattributed ramifications in *white*. The scale bar equals 50 μm. **c** The morphological criteria to characterize a microglial cell. The cell body detection (*blue*) and cytoplasm area (*pink*) have performed as a starting point to characterize a microglial cell. The complexity index (*green*) and the covered environment area (CEA in *orange*) have been deduced from ramification detection. The scale bar equals 10 μm. **d** Two-dimensional cartography at a single cell resolution. *Colours* correspond to the range of complexity and CEA with a gradient from a low level of complexity and CEA (*yellow*) to a high level of complexity and CEA (*red*). The scale bar equals 50 μm. For illustration, the images are contrast adjusted to aid in visualizing the GFP expression

Before the shape characterization analysis, focal stacks of each mosaic were reconstructed by combining images from the different focal depths. Each stack was subsequently divided into three 10-μm sub-volumes to allow a two-dimensional (2D) maximum intensity projection analysis (Fig. 1a), consistent with the average size of cell bodies. Mosaic, volume creation and maximum projection processing from confocal images were done using automated free plugins [32] of the ImageJ v1.50 software interface [33].

### Image analysis for characterization of microglial cell population

An automatic image analysis was performed consecutively on the three maximum projection mosaics described above, using a custom-designed script developed with the Acapella™ image analysis software (version 2.7, PerkinElmer Technologies, Waltham, USA). This script was subdivided into two subroutines: the first, for automated detection of processes (neurite detection module from Acapella™ [34]), generating morphological characteristics per cell, and the second, for the 2D in situ morphological cartography. The data workflow is illustrated step by step in Additional file 1.

### Microglial morphological criteria

Using the custom-designed script cited above, the following morphological criteria could be extracted for each microglial cell (Fig. 1b, c): a set of measured criteria as cell body and cytoplasm area, defined as the cell body area associated with the cytoplasmic area of the primary ramifications, expressed in μm^2^; branching characteristics such as the total number and length (μm) of ramifications and the number of primary, secondary and tertiary ramifications; and roundness (ratio between surface and perimeter squared of the cell body) and GFP intensity by whole cell.

A second set of calculated criteria extrapolated from the previous ones yielded the complexity index (CI) and the covered environment area (CEA). First, we defined the CI using two different criteria extracted from the Acapella™ script: the number of segments of each cell, a segment being defined as the length of process between two nodes, and the number of its primary ramifications. By dividing these two criteria, we obtain also a mean complexity by primary ramification for each microglial cell (Additional file 2). On the other hand, CEA represents the 2D total surface covered by its ramifications and defined as the area of the polygon formed by linking the extremities of its processes, expressed in μm^2^. The areal density of microglial cells by region or by brain was calculated by dividing the number of microglial cells selected by the scanned tissue area.

The CI revealed a completely distinct microglial phenotype, the *amoeboid cells*. The amoeboid or rod cells are characterized by a CI = 1 (no nodes) and are characteristic for activated cells, displaying engulfing, phagocytic properties [11]. Because of their particular role, we distinguish them from the other microglial cells.

### Elimination of outliers and redundant cells

We proceeded to the filtration of outliers by size (inferior to 10 μm^2^ or superior to 500 μm^2^) and roundness (inferior to 0.7 considered as the limit of a noisy form) to eliminate artefacts due to tissue noise. Using the property of our Acapella™ script to generate in situ 2D cartographies (Additional file 1), cell duplicates on two adjacent sub-volumes were rejected for analysis to avoid data duplication. This 2D localization in situ allowed us also to remove the cells located at the edges of the mosaic reasoning that they may be truncated.

### Manual and semi-automated method workflow

To assess the accuracy of the 2D reconstruction of the custom-designed script cited, we compared the criteria measured by the cell using our automated method with the analysis realized manually by three independent experimenters, as a technical benchmark: the cell body area, the number of segments, the number of primary ramifications and the measure of the CEA. The manual method has been performed using Fiji environment (Additional file 3).

In parallel, to assess that the criteria analysis is not software specific, a free semi-automated method has been implemented in a Fiji macro using Skeletonize (2D/3D) and Sholl Analysis plugins [35, 36]. The cell body area, the number of segments and the number of primary ramifications have been extracted by this custom-designed macro.

These two methods were tested and compared with our automated method in two regions of interest, the hippocampus and the cerebellum, and in the two conditions, LPS and control.

### Data analysis and statistics

All the data extracted from Acapella™ after elimination of outliers and redundant cells were exploited following two levels: (i) by the brain and (ii) by specific region. We conducted this study using two different statistical methods. The first conventional method consisted in an approach considering only the median or the average for each morphological criterion by animal. The second approach, more powerful and original, presented results using *k-*means clustering by cell population.

To realize the conventional approach, Prism 6.0 (GraphPad Software Inc.^©^, USA) was used for statistical analysis by animal and regions of interest. Data were analysed via Mann-Whitney test or Student’s *t* test after being assessed for normality of sample distribution. Inter-sample/inter-region variability was tested by ANOVA Kruskal-Wallis method. Qualitative traits (i.e. clustering phenotype distribution) were analysed with a chi-square (*χ*^2^) test. Spearman coefficient has been expressed to represent the correlation between two sets of data. Statistical significance is shown on the graphs (*p < 0.05; **p < 0.01; ***p < 0.001; ****p < 0.0001). Statistical tests used for each data set are indicated in the figure legends.

The required number of samples per group (*n*) has been evaluated with pwr.t.test R function [37], with *α* = 5 % and 1 − *β* = 90 %.

### The clustering analysis

In a second time, Prism data were transferred into JMP® version 11.0 (Statistical Analysis System Institute Inc., USA) for a complete multivariate analysis by cell population. A principal component analysis (PCA) was performed to identify the correlation between the different analysed features. To detect and characterize the sub-population of microglial cells, a *k-*means clustering method, appropriated for a large set of data, has been applied (*k* = 4). The statistics of each cluster (mean and frequency) were used to characterize sub-populations and determine their phenotype, later named clusters 1 to 4. For each condition, the amount of microglial cells analysed was about 810 by region, 2870 by brain or 20,000 by group.

### Data storage and annotations by in situ 2D cartography

All acquired and analysed mosaics have been imported into the OMERO (“OME Remote Objects technology”) image database [38] including visual results in 2D cartographies by phenotyped cell (Fig. 1d and Additional file 1). An open-source script (OMERO.csv) has been used to annotate automatically by textual information (i.e. sex, condition or clinical observation by sample or region appurtenance) our large set of imported images in the database.

## Results

Using data extracted from more than 20,000 cells per condition, we performed and compared two types of complementary statistical approaches at an inter-regional and an inter-group level. To validate the acquisition process, the GFP intensity of each microglial cell was measured. No difference in the microglial cell GFP intensity between the two conditions, whatever the brain region, was found (Table 1 and Additional file 4).

**Table 1:** Morphological variability study for microglial cells between two groups

Criteria	H	FC	S	C
GFP intensity	0.2331	0.5245	0.5245	0.4394
Cell body area	*0.0221*	*0.0221*	*0.0140*	*0.0012*
Cytoplasm area	*0.0012*	*0.0012*	*0.0012*	*0.0082*
Complexity	0.1364	0.6154	0.6037	*0.0012*
CEA	0.9172	0.2925	0.6154	0.1783
Density	0.0734	0.9749	0.9172	0.1014

Values are expressed as Mann-Whitney exact *p* values; significant differences in italics

### Conventional statistical approach

**Fig. 2:**
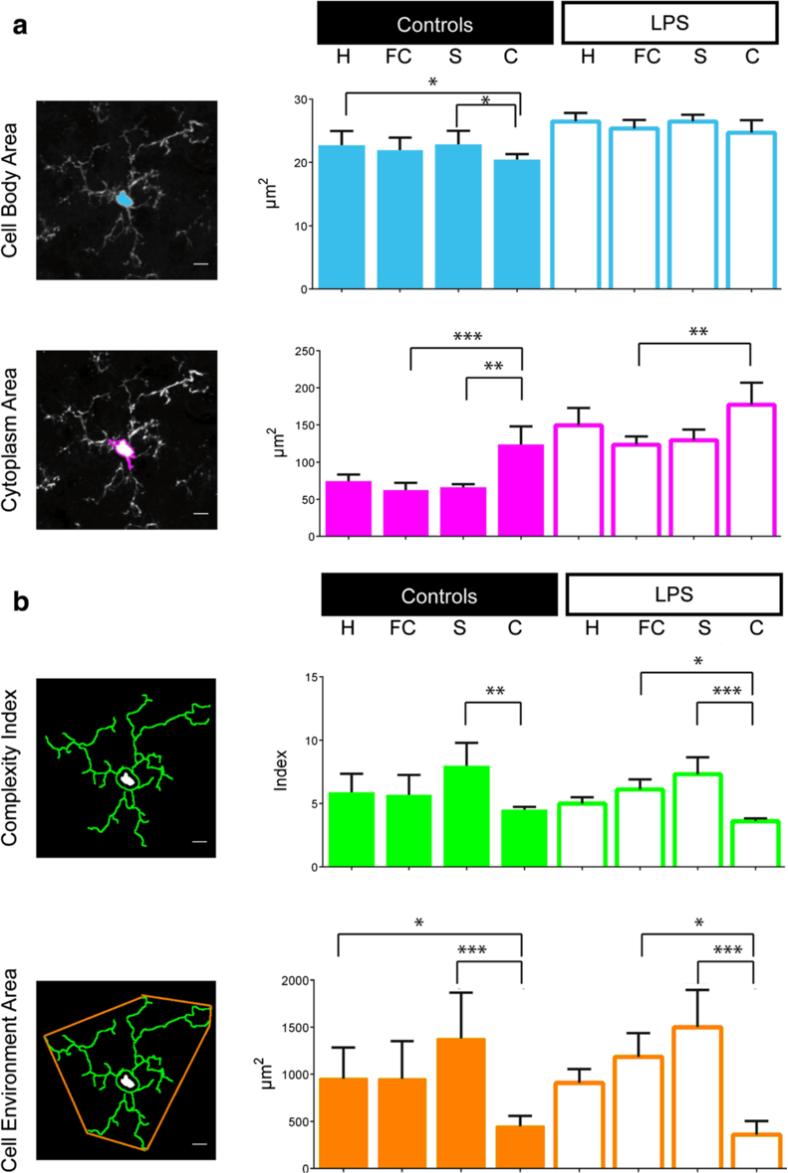
The inter-region variability by morphological criteria. Four regions have been explored: hippocampus (*H*), frontal cortex (*FC*), striatum (*S*) and cerebellum (*C*) in two different conditions, the control (*left column*) and the LPS (*right column*). **a** Historical parameters to characterize the microglial morphology: the cell body area and the cytoplasm area defined as the cell body area associated with the cytoplasmic area of the primary ramifications in μm^2^. **b** Calculated criteria extrapolated from the Acapella™ script: the complexity index (CI) and the covered environment area (CEA), in μm^2^. Data shown are means ± SD in the control and LPS groups (*n* = 7 and *n* = 6, respectively). The scale bars equal 10 μm. ANOVA Kruskal-Wallis test was used to compare the different regions. *p < 0.05, **p < 0.01, ***p < 0.001

**Fig. 3:**
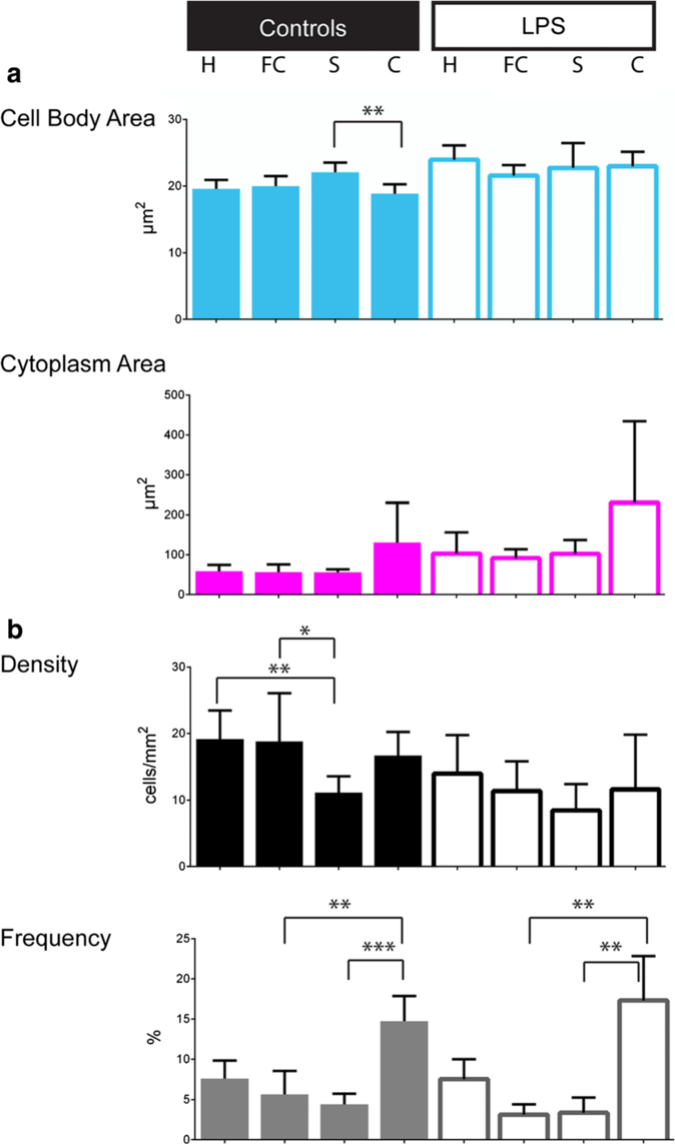
Characteristics of the amoeboid population and their inter-region variability. Bar charts represent the characteristics of the amoeboid cell morphology (characterized by a CI = 1, without nodes) and the distribution within the four explored regions: hippocampus (*H*), frontal cortex (*FC*), striatum (*S*) and cerebellum (*C*) in the two different conditions, the control (*left column*) and the LPS (*right column*). **a** Parameters to characterize the amoeboid cells morphology: the cell body area and the cytoplasm area defined as the cell body area associated with the cytoplasmic area of the primary ramifications in μm^2^. **b** Parameters to characterize the amoeboid cell distribution: density calculated by dividing the number of microglial cells selected by the scanned tissue area (3.03 mm^2^) and frequency as the ratio between the number of amoeboid cells and the total number of microglial cells analysed. Data shown are means ± SD per condition (*n* = 7, *n* = 6 for control and LPS, respectively); we used ANOVA Kruskal-Wallis test. **p < 0.01, ***p < 0.001

For this conventional approach, two statistical analyses were performed: one to observe an inter-region variability (Figs. 2 and 3), the other to detect a difference inter-group by brain region (Tables 1 and 2).

**Table 2:** Morphological variability study for amoeboid between two groups

Criteria	H	FC	S	C
GFP intensity	0.7133	0.9021	0.7133	0.8135
Cell body area	*0.0023*	0.1014	0.8135	*0.0082*
Cytoplasm area	*0.0221*	*0.0221*	*0.0012*	0.1375
Density	0.1288	*0.0221*	0.1014	0.0734
Frequency	0.9021	0.2308	0.1276	0.5058

Values are expressed as Mann-Whitney exact *p* values; significant differences in italics

#### Microglial cell body area, cytoplasm area and density

Mean cell body and cytoplasm areas of microglial cells in the LPS group brains are significantly higher than in the control group brains (respectively, 149 vs. 74 μm^2^ for the cytoplasm area in the hippocampus, *p* = 0.0012, Table 1). We found a high heterogeneity between the mean microglial cell body and cytoplasm areas in the different regions of the brain within a given group (*p* < 0.01 or *p* < 0.001). At the opposite, in the context of LPS-induced inflammation, the mean cell body area was homogeneous wherever the region (Fig. 2a). Moreover, in an inter-regional comparison, the mean cytoplasm area was significantly greater in the cerebellum than in the other regions, in either the control or LPS groups (Additional file 4); it did not statistically differ between the hippocampus, frontal cortex and striatum, in both conditions. In an inter-group comparison (Table 1), we observed a significant increase of cell area (body and cytoplasm) in the LPS group; the microglial cell cytoplasm was twice greater after the LPS challenge regardless of the region. In each condition, the microglial density varied significantly among regions, with a higher mean density in the frontal cortex and a lower one in the cerebellum (Additional file 4). The microglial density did not differ between the control and LPS groups, in the total brain or in each region (Table 1).

#### Complexity index (CI) and covered environment area (CEA)

Both microglial CI, defined as the mean complexity by primary ramification, and CEA, defined as the 2D total surface covered by microglial ramifications, showed no statistical difference between the two different groups. CI and CEA were significantly lower in the cerebellum than in the other regions, in both the control and the LPS group. We did not find any statistical difference between the hippocampus, frontal cortex and striatum regardless of the conditions (Fig. 2b). CI and CEA did not statistically differ between the LPS and control groups, in each region except the cerebellum (Table 1). The cerebellar microglial CI was significantly lower in the LPS group than in the control group (Additional file 4).

#### Comparison with Iba-1 expression based on morphological criteria by sample

To strengthen our statements about the CX3CR1^GFP/+^ mice model, we performed the same experiments based on Iba-1 expression in wildtype C57BL/6 mice and we were interested in the same morphological criteria (microglial cell body area, cytoplasm area, cytoplasm intensity, CI and CEA). In the hippocampus, we observed the same differences between the two groups compared to the GFP model with a significant increase of cell area (body and cytoplasm) in the LPS group whereas we observed no difference considering the CI and CEA (Additional file 5). In the cerebellum, despite a trend comparable to what is seen in the GFP mouse model, there is no significant difference in the cell area. This isolated difference may be explained by the smaller number of cells analysed, about 10 times less, linked to the limits of the immunostaining on thick sections.

#### Amoeboid cells

In each condition, the frequency of amoeboid cells, considered as a particular group of microglial cells with specific morphological characteristics (CI = 1) associated with a specific function, varied significantly among regions, with the lowest frequency in the frontal cortex than in the cerebellum (Fig. 3): control group: 5.64 ± 2.9 % and 14.73 ± 3.11 %, respectively, *p* = 0.0078; LPS group: 3.13 ± 1.27 % and 17.30 ± 5.53 %, respectively, *p* = 0.0018 (Additional file 4). The LPS challenge was associated with increased amoeboid cell body and cytoplasm areas among brain regions. There is no difference by region in terms of amoeboid frequencies between the two groups (Table 2 and Additional file 6).

In conclusion, using a conventional statistical approach, we found that (i) in comparison to other regions, cerebellar microglial cells presented a bigger cytoplasm, were less dense and complex, more frequently amoeboid and more responsive to LPS; (ii) the LPS challenge is associated with an increase, such as twice greater, in cell body and cytoplasm areas but not with a decrease in CI and CEA, indicating that there is no evidence for a “deramification” process.

### Comparison with benchmark methods

To assess the accuracy of our automated method, after random extraction of an analysed cell subset, three independent experimenters performed manual measures of the selected criteria as cell body area, CI and CEA. Statistical tests carried out by region showed the same trends between the control and LPS groups whatever the method considered (Additional file 7A). It is to be noted that the analysis time per cell is multiplied by 10 between the automated method and the manual method and the number of cells studied by condition is 400 times lower (Additional file 7C).

We also tested whether the results of the analysis were not software specific. Using a semi-automatic analysis with the Fiji software environment on a greater number of cells than in the manual comparison, we did not find any difference in the morphological criteria between the semi-automatic and automatic methods in each region. The results obtained showed a strong correlation between the two methods (Additional files 7B, C).

### Cell heterogeneity by condition

Although no difference was observed between the mean CI or CEA of the microglial cells in the two conditions, considering every cell of every brain in each condition, we found a high cell heterogeneity between the samples using Kruskal-Wallis test (*p* < 0.0001) illustrating the biological variability across individuals (Additional file 8), whatever the morphological criteria tested.

Such variability does not allow us to compare directly populations using classical statistical studies. Moreover, in order to distinguish sub-populations in our large datasets of cells, we pooled all the microglial cells from each group by region and also conducted a new statistical study using a clustering method.

### Statistical clustering approach

**Fig. 4:**
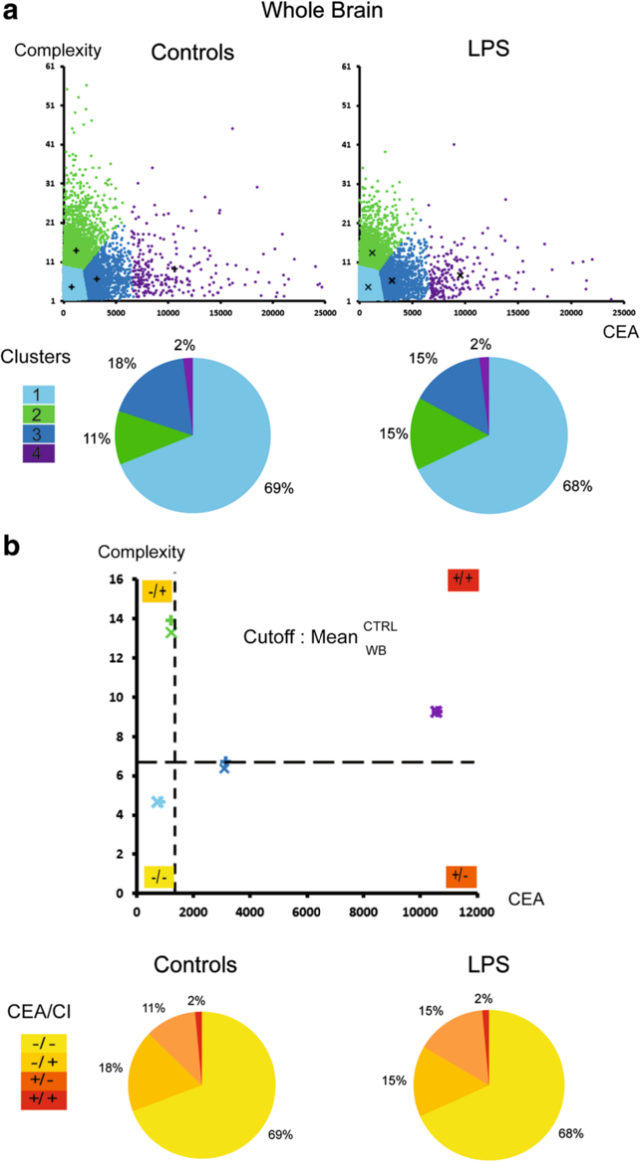
Approach by clustering to track sub-populations of microglial cells in the whole brain. **a** The scatter plots illustrate, at a single cell resolution, the CEA and CI characteristics and their frequency by cluster. The symbols “+” and “x” correspond to the centre of each cluster by the control and the LPS condition, respectively. The pie charts show the cluster frequencies by *k*-means clustering method (*k* = 4), and no significant difference has been observed between the two conditions using the chi-square test. **b** Four sub-populations have been defined by the cutoff (*dotted lines*) fixing the high (+) or the low (−) characteristic of one sub-population in the whole brain (*WB*). The cutoff was defined as the average of each morphological criterion (CI and CEA) in the control group. The centre of each cluster was plotted in the graph. The pie charts represent the proportions of sub-populations by condition. The same repartitions by sub-population as by cluster were observed

**Fig. 5:**
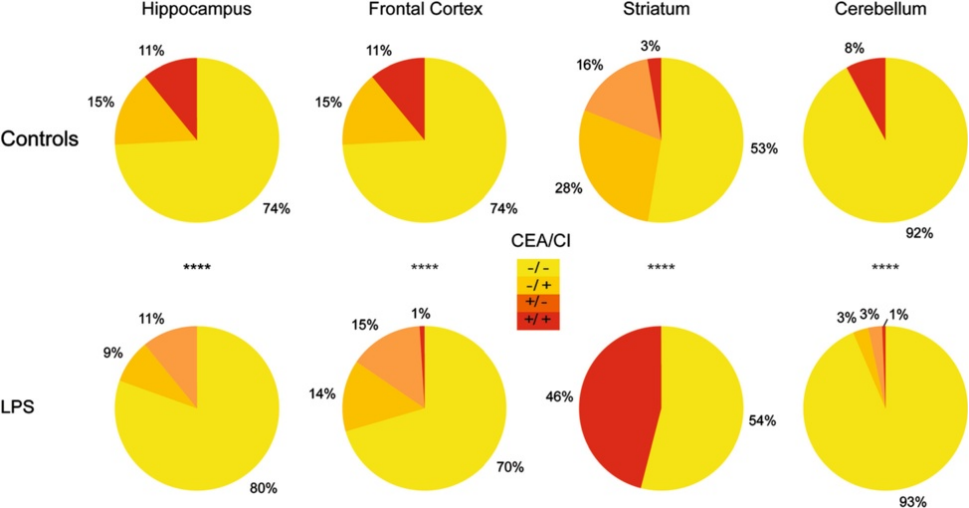
Highlighting sub-populations by region. The pie charts represent the proportions of sub-populations defined by the cutoff previously described in Fig. 4 by region of interest and by condition: in *yellow*, sub-population with low CEA and low CI (−/−); in *orange*, sub-population with low CEA and high CI (−/+); in *dark orange*, sub-population with high CEA and low CI (+/−); and in *red*, sub-population with high CEA and high CI (+/+). Chi-square test was used to compare the control and the LPS group. ****p < 0.0001

The principal component analysis (PCA) showed that the CI and CEA did not correlate, allowing proceeding to the cluster analysis based on these indexes. In the control group, based on the whole brain without region discrimination, the rates of the clusters 1, 2, 3 and 4 used to characterize sub-populations by the *k*-means clustering were 69, 18, 11 and 2 %, respectively (Fig. 4a). The cutoff fixing the high or the low characteristic of one population was set as the average of each morphological criterion in the control group (Fig. 4b) and therefore allowed to discriminate four sub-populations (SP): SP1: low CEA and low CI (−/−); SP2: low CEA and high CI (−/+); SP3: high CEA and low CI (+/−); and SP4: high CEA and high CI (+/+). Considering the whole brain, the proportions of SP did not differ statistically between the control and LPS conditions (Fig. 4b). A contrario, the proportions of these sub-populations varied significantly among the regions in the control groups, as shown in Fig. 5. SP1 was most represented in the cerebellum (92 % of the cells). SP2 represented from 28 % of the cells in the striatum to 15 % in the hippocampus. SP3 is present uniquely in the striatum at a 16 % rate and SP4 from 3 % in the striatum to 11 % in the hippocampus. The regional distribution of the sub-populations varied significantly between the control and LPS conditions (*p* < 0.0001), with a significant increase in the SP4 in the striatum (from 3 to 46 %) and at the opposite a significant decrease to disappearance of this population in the other areas. The SP3 increases largely in the frontal cortex, hippocampus and cerebellum. The SP1 remains stable except in the hippocampus with an increase up to 80 % of the cell population (Fig. 5).

Cluster analysis based on CI and CEA revealed a regional pattern of microglial sub-population with particular responsiveness to LPS. Therefore, we obtained a new frequency of phenotyped cells and hereby defined sub-populations of microglial cells based on particular behaviour.

### Impact on the number of needed animals

The impact of a high-content automated approach is also important ethically. It is possible to assess the number of mice required to highlight the same differences as those observed using a classical statistical study and considering only the mean by animal. This evaluation of the number of subjects required (*n*) is based on the two-sample *t* test defined by the following formula:

$$ n=2\times t\times \frac{\sigma^2}{{\left({\overline{m}}_A-{\overline{m}}_B\right)}^2} $$

in which ${\overline{m}}_{A,B}$ are the mean of criteria for two sets of data and *σ* is the common standard deviation of two samples with the hypothesis that *n*_*A*_ = *n*_*B*_.

Considering a power of 90 % and an alpha risk of 5 %, the expected sample size showing exactly the same differences is from 1 to 27 times greater considering the CI depending on the brain area or from 6 to 100 times greater considering the CEA. This sample size is summarized in Table 3.

**Table 3:** Expected sample size to obtain significant differences between the two groups using a conventional approach

	${\overline{m}}_{\kern0.75em \mathrm{CTRL}}\left({\overline{m}}_{\mathrm{LPS}}\right)$	*σ*	*n*	${\overline{m}}_{\kern0.75em \mathrm{CTRL}}\left({\overline{m}}_{\mathrm{LPS}}\right)$	*σ*	*n*
	H			FC		
CI	5.9 (4.9)	1.2	36	5.7 (6.1)	1.2	189
CEA (μm^2^)	953 (908)	253	643	950 (1184)	349	47
Cytoplasm area (μm^2^)	74.4 (149.4)	42.2	7	62.4 (123.0)	33.0	7
	S			C		
CI	7.9 (7.3)	1.6	114	4.5 (3.6)	0.5	7
CEA (μm^2^)	1378 (1499)	435	272	450 (360)	130	45
Cytoplasm area (μm^2^)	66.3 (129.2)	34.1	7	123.4 (177.0)	37.9	11

Where $\overline{m}$ is the mean of criteria for the control (LPS) set of data, *σ* is the common standard deviation of the two groups and *n* is the expected number of samples to obtain a significant difference

## Discussion

Microglial morphological analysis is the historical technique to describe the microglial cells and also the only way to study these cells within the complex environment of the central nervous system [39]. In the literature, four major microglial phenotypes are usually distinguished based on distinct morphological criteria [11]: ramified (presenting a small cell body and numerous branched ramifications) that constitute about 90 % of the microglial pool between 1 and 3 months in the murine model and are considered to be the microglial “resting” state; primed (bigger cell body but unchanged ramification pattern compared to ramified phenotype); reactive (even bigger cell body, shorter, fewer and thicker ramifications); and amoeboid (two or less processes without any branch). Despite a probable link between morphology and function [40–42] and due to a large panel of slight microglial morphological changes [28], morphological analyses are subject to many hurdles, limiting their strength in terms of objectivity, precision and accuracy leading to subjective interpretations.

The objective of the present study was to develop a new powerful tool and method for describing microglial morphology and for assessing its variability among brain regions in order to further characterize and understand its behaviour in the context of development, homeostasis and disease. We confirmed the added value of our method by studying the changes related to the LPS challenge, a well-characterized model to study inflammation. This method is based on a clustering analysis integrating new developed and automatically acquired morphological indexes, the complexity index (CI) and the covered environment area (CEA), the area covered by microglial ramifications. These two criteria are critical since different experimental studies [43–45] highlighted certain kinetics not only in morphological changes after microglial stimulation including rapid microglial process growth, extension and reorientation towards injury but also in response to healthy central nervous system (CNS) environment [46–48].

For assessing the discriminatory power of our approach, we compared the clustering analysis to parametric and non-parametric statistical approaches. We found that the clustering analysis allowed us to identify different microglial phenotypes that are heterogeneously distributed among brain regions and presenting different behaviours under the LPS challenge. The parametric statistical tests failed to identify these sub-populations.

As a first step, we compared available methods that are manual, qualitative or semi-quantitative to ours. Differences arise in (i) a number of cells analysed 30 times greater than reported by previous studies [25–27], (ii) an objective quantification of the morphological parameters providing a reliability in numerical and resolving power and (iii) providing a significant savings in time with an analysis that is 10 times faster. Second, while the CX3CR1^GFP/+^ transgenic model is a standard tool to study microglial morphology in in vivo [49, 50] or ex vivo experiments [51, 52], we performed the same experiments based on Iba-1 expression in C57BL/6 mice and observed the same trends than those observed with the GFP model considering the main morphological criteria. Nonetheless, this approach allows to analyse 10 times less cells than the CX3CR1^GFP/+^ thanks to the immunohistochemistry techniques, confirming the interest of the use of a knock-in model.

The relevance of CI and CEA indexes, easily assessed with automated methods, is confirmed by their ability to identify morphological sub-types with a particular regional distribution and sensitivity to LPS. The lack of collinearity between these two derived indexes indicates that they provide specific morphological information that may reflect particular functional status. One may argue that for a given covered area, hypo- and hyper-ramified microglial cells have different roles.

The last methodological input concerns the analysis by *k*-means clustering, considered to be a simple but efficient algorithm to define sub-populations of identical phenotyped cells. It divides the data into *k* clusters, minimizing the squared distance between each data point to the centre of its cluster. The main interests of this algorithm are its speed and ease of interpretation, and it is particularly adapted for identifying sub-groups in a large dataset by cellular heterogeneity recognition [53]. The principal limitations are that it requires an a priori specification of the number of cluster centres and has a strong sensitivity to outliers and noise. Together with Kongsui and colleagues [54] in a recent study interested in the structural alterations of the microglial cells within the prefrontal cortex in rats following LPS injection, we do not find any statistical difference in the mean values of CI or CEA. Kongsui and colleagues raise the hypothesis (i) that microglial process alteration is a later phenomenon or (ii) that a substantially larger group of cells studied associated with improved analytical approaches may reveal differences. The results of our cluster analysis based on these indexes that identify major changes in the global and regional distribution of morphological sub-types support the second hypothesis. This discrepancy clearly illustrates the discriminating power of cluster analysis, when compared to parametric tests.

The regional heterogeneity of microglial morphology and the microglial effect of the LPS challenge have long been studied [1, 55]. In our study, we confirm this regionalization of the microglial distribution with a decrease in density, CI and CEA indexes from the frontal cortex to the cerebellum. Injection of LPS was associated with an increase in the cytoplasmic area and in the proportion of amoeboid cells. Kozlowski and Weimer found the same trend into the cortex in their study in 2012, correlated with an overexpression of Iba-1 and CD68 [24]. In a more original way, we observed a regional susceptibility to LPS thanks to the microglial morphology, as it is also observed at a protein level by various experimental studies [56, 57]. This regionalization of pathophysiological processes at cellular and proteic levels supports the clinical and behavioural specific responses to neurological challenges [58, 59]. A method like ours may contribute to assess the nature of the microenvironmental factors involved in microglial shape and reactivity [60, 61].

Since the cluster analysis enables the assessment of a large population of cells per animal, it dramatically reduces the number of animals needed for testing a hypothesis. For instance, it would have been necessary to sacrifice 6 to 100 times more mice to observe a statistical difference in CI (or CEA) between the LPS- and non-LPS-treated groups, using a parametric test. This is a major ethical advantage and in compliance with the European and American requests.^1^ Thanks to the large number of cells analysed in the same animal with our automated method, it is also possible to use an accurate statistical approach and consequently to dramatically improve the ethical considerations of experimental works.

Although we have found and confirmed the heterogeneity of microglial cells both in the resting and the inflammatory brain, a functional assay to correlate the morphology with function is still required. Many markers have been described to characterize different microglial activation stages, but the use of these markers only would not have been sufficiently precise, and more parameters are thus needed to further link morphology and microglial function. However, our results further confirm data from the literature as Kozlowski and Weimer showed it [24].

One of the strengths of this approach is that other functional or morphological parameters could be included in the future according to the focus of the experimenters to highlight different behaviour or improve the precision of the study. It is also possible that increasing the number of parameters could lead to the discovery of novel microglial cell states before, during and after pathological conditions.

Moreover, our technique benefits from the ease of accessibility and will allow all labs to use these parameters as a tool to characterize the microglial behaviour and further understand in a standardized manner its role in healthy and diseased condition.

Two main fields could benefit from this approach. First, through the use of optical sectioning microscopy, it is possible and easy with our method to work on a three-dimensional network. The appearance of clusters of complex cells or of similar activation states may confirm what is already highlighted in 2D or, on the contrary, may reveal new behaviours. Second, in subsequent studies, it would be useful to correlate the precise morphology of a cell that we are able to identify with its function, possibly using single cell techniques in situ after morphological analysis within tissue.

## Conclusions

Our study presents an automated approach of morphological analysis coupled with a high-content statistical study that allows highlighting sub-populations of microglial cells, even in a healthy condition, counteracting the classical view of a homogenous resting state. These sub-populations present specific behaviour after neuroinflammatory challenge induced by LPS, allowing postulating a cellular specialization specified by its morphology. Moreover, our study confirms the need to work region by region considering this type of cells. This statistical approach may become the cornerstone of any study involving dendritic-shaped cells while seeking the reduction of the number of animals in accordance with the international guidelines for animal welfare.

## Abbreviations

CEA, Covered environment area; CI, Complexity index; CNS, Central nervous system; GFP, Green fluorescent protein; Iba-1, Ionized calcium binding adapter molecule 1; LPS, Lipopolysaccharide; OMERO, OME Remote Objects technology; PCA, Principal component analysis; SP, Sub-population

## Additional files

Additional file 1: High-content analysis workflow overview, from acquisition to statistics for the seizure of a large number of brain regions of mice, their classification and associated statistical analysis. Acquisition pipeline: four regions of interest were scanned using a spinning disc confocal system (CV1000-Yokagama): striatum (S), frontal cortex (FC), hippocampus (H) and cerebellum (C) representing approximately 13 mm^2^ of the entire brain surface with a depth of 30 μm. The voxel size is equal to 0.19 × 0.19 × 2.0 μm^3^, respectively, for the *X*, *Y* and *Z* dimensions by stack. Analysis pipeline: using a macro implemented in ImageJ free software, each data stack was divided into three sub-volumes followed by a maximum projection for two-dimensional (2D) analysis. Each sub-volume (v1 to v3) was analysed by Acapella^TM^ software to extract morphological criteria for each microglial cell. The original image data and generated in situ 2D cartographies were stored in Image Database for visualization, sharing and clinical annotations. At the end, statistics were done with the data extracted from this analysis pipeline. (TIF 1480 kb) Additional file 2: The complexity index as new morphological criteria. The complexity index (CI) for each microglial cell was defined by dividing two different criteria: the number of segments of each cell and the number of its primary ramifications. (A) A typical schematic microglial cell presenting a circular cell body area (in red) and some ramified processes. Each of them is composed of one primary ramification (white arrow) and several sub-ramifications separated by nodes (blue arrows). One segment is defined as the length of process between two nodes. Each segment is visible in a single colour. (B) In the left column, individual microglia based on GFP fluorescence appears in white outline. In the right column, schematic representation of the microglial cell characterized by its CI (white text). The scale bars equal 10 μm. (TIF 676 kb) Additional file 3: Data analysis workflow with manual and semi-automated methods. The manual method has been performed using Fiji environment and selection drawing tools to measure the cell body area, the number of roots, ramifications and the CEA. The semi-automated method has been implemented in a Fiji macro using successively Analyze Particles, Skeletonize (2D/3D) and Sholl Analysis plugins. The scale bars equal 10 μm. (TIF 1987 kb) Additional file 4: Descriptive statistics for microglial cells by condition. (PDF 13 kb) Additional file 5: Characterization of microglial cells by morphological criteria based on Iba-1 expression. Two regions have been explored: hippocampus (H) and cerebellum (C) in the control or LPS conditions. The scatter plots illustrate, by analysed cell, the body area (in blue), cytoplasm area (in pink) and intensity (in grey), CI (in green) or CEA (in orange) characteristics for each animal in both groups. Data shown are means ± SD. The scale bars equal 10 μm. The Mann-Whitney test was used to compare the control and LPS groups (respectively, *n* = 5 and *n* = 6).**p* < 0.05. (TIF 262 kb) Additional file 6: Descriptive statistics for amoeboid cells by condition. (PDF 53 kb) Additional file 7: The comparison of different quantitative analysis methods for microglial morphological criteria by cell. (A) The scatter plots illustrate, at a single cell resolution, the body area, CI or CEA characteristics for the automatic or manual method in the control and LPS groups. The Mann-Whitney test was used to compare the control and LPS groups (respectively, *n* = 5 and *n* = 6).**p* < 0.05. (B) The correlation plots of two morphological criteria between semi-automatic and automatic analysis with cell body area (in blue) and CI (in green) at a single cell resolution. Values indicate the Spearman correlation coefficient. The lines represent the linear regression. (C) The table indicates the number of cells (Nb cells), the cell body area, the CI, the CEA and the mean analysis time per cell for each condition (control and LPS groups) and each region (hippocampus and cerebellum). (TIF 424 kb) Additional file 8: Quantitative analysis of microglial cell morphology in the whole brain considering CI and CEA. The scatter plots illustrate, at a single cell resolution, the CI or CEA characteristics for each animal in both groups. ANOVA Kruskal-Wallis was used to test the inter-sample heterogeneity. Data shown are means ± SD. *****p* < 0.0001. (TIF 438 kb)

## Footnotes

1 Directive 2010/63/EU of the European Parliament and of the Council.
