# Supplementary figures and images for: Phenotypic clustering: a novel method for microglial morphology analysis
Source: J Neuroinflammation. 2016 Jun 17;13:153. doi: 10.1186/s12974-016-0614-7 (PMC4912769; doi:10.1186/s12974-016-0614-7)

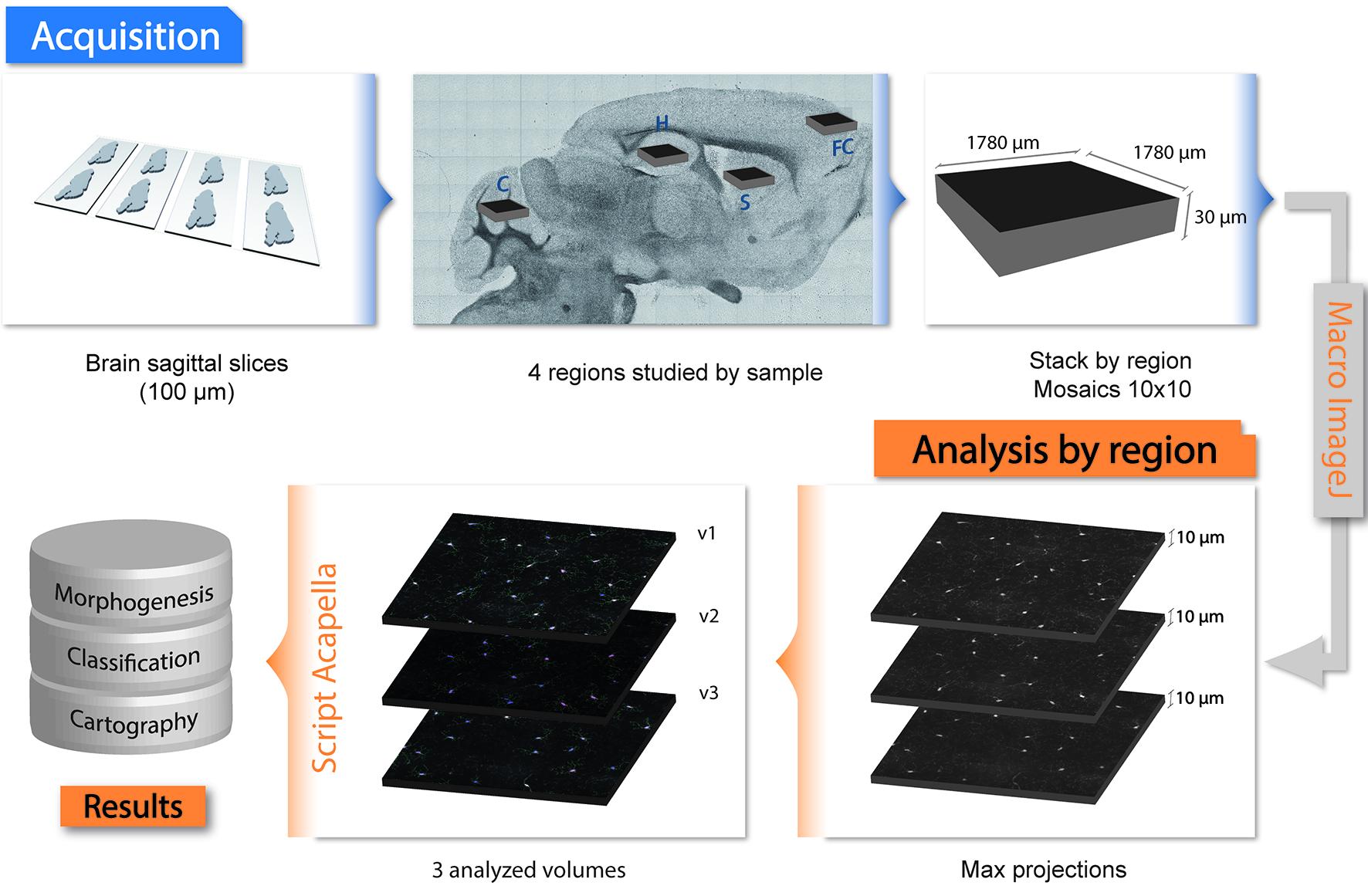

Supplement: Additional file 1: — High-content analysis workflow overview, from acquisition to statistics for the seizure of a large number of brain regions of mice, their classification and associated statistical analysis. Acquisition pipeline: four regions of interest were scanned using a spinning disc confocal system (CV1000-Yokagama): striatum (S), frontal cortex (FC), hippocampus (H) and cerebellum (C) representing approximately 13 mm2 of the entire brain surface with a depth of 30 μm. The voxel size is equal to 0.19 × 0.19 × 2.0 μm3, respectively, for the X, Y and Z dimensions by stack. Analysis pipeline: using a macro implemented in ImageJ free software, each data stack was divided into three sub-volumes followed by a maximum projection for two-dimensional (2D) analysis. Each sub-volume (v1 to v3) was analysed by AcapellaTM software to extract morphological criteria for each microglial cell. The original image data and generated in situ 2D cartographies were stored in Image Database for visualization, sharing and clinical annotations. At the end, statistics were done with the data extracted from this analysis pipeline. (TIF 1480 kb) [file 12974_2016_614_MOESM1_ESM.tif]

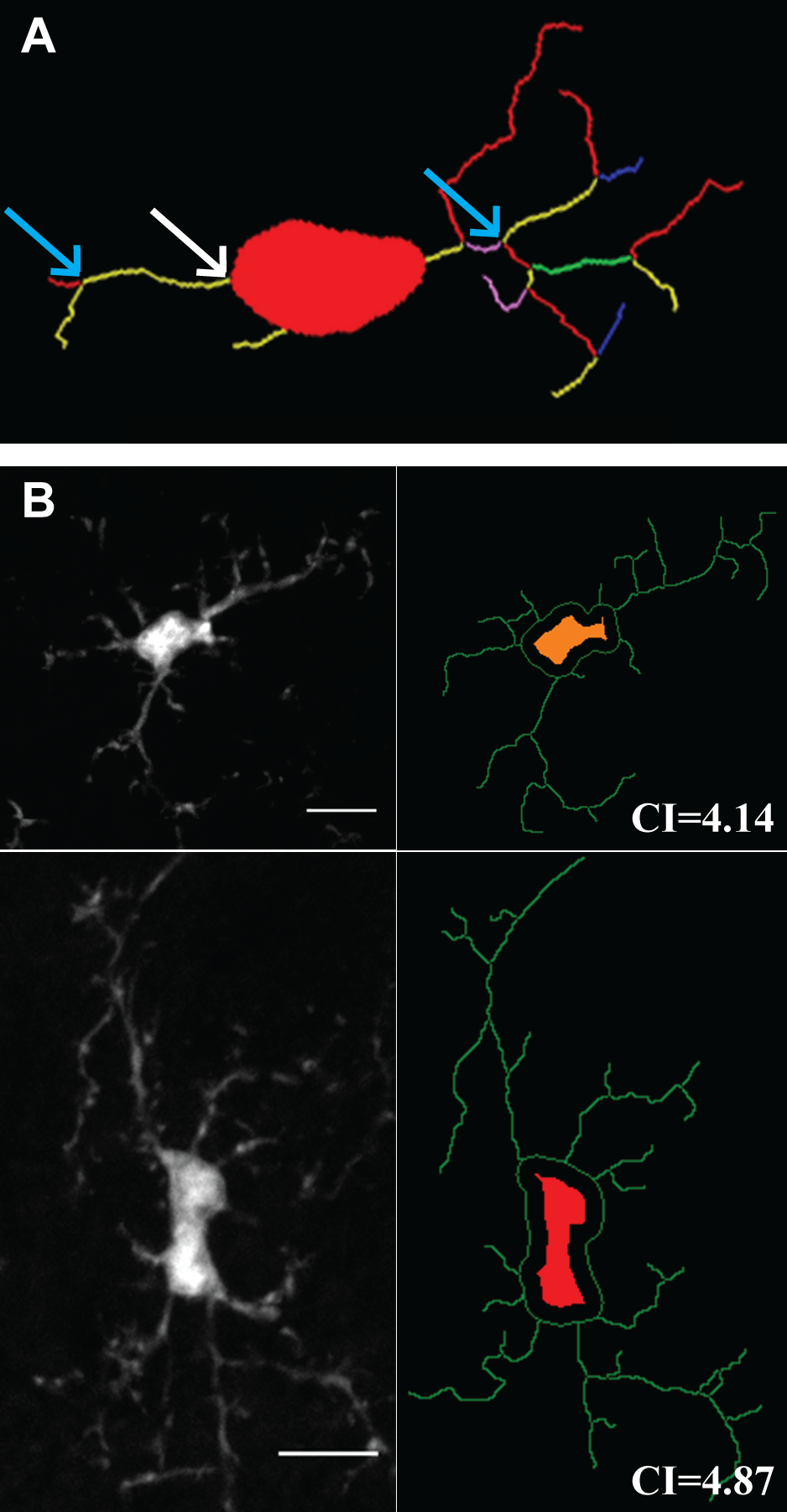

Supplement: Additional file 2: — The complexity index as new morphological criteria. The complexity index (CI) for each microglial cell was defined by dividing two different criteria: the number of segments of each cell and the number of its primary ramifications. (A) A typical schematic microglial cell presenting a circular cell body area (in red) and some ramified processes. Each of them is composed of one primary ramification (white arrow) and several sub-ramifications separated by nodes (blue arrows). One segment is defined as the length of process between two nodes. Each segment is visible in a single colour. (B) In the left column, individual microglia based on GFP fluorescence appears in white outline. In the right column, schematic representation of the microglial cell characterized by its CI (white text). The scale bars equal 10 μm. (TIF 676 kb) [file 12974_2016_614_MOESM2_ESM.tif]

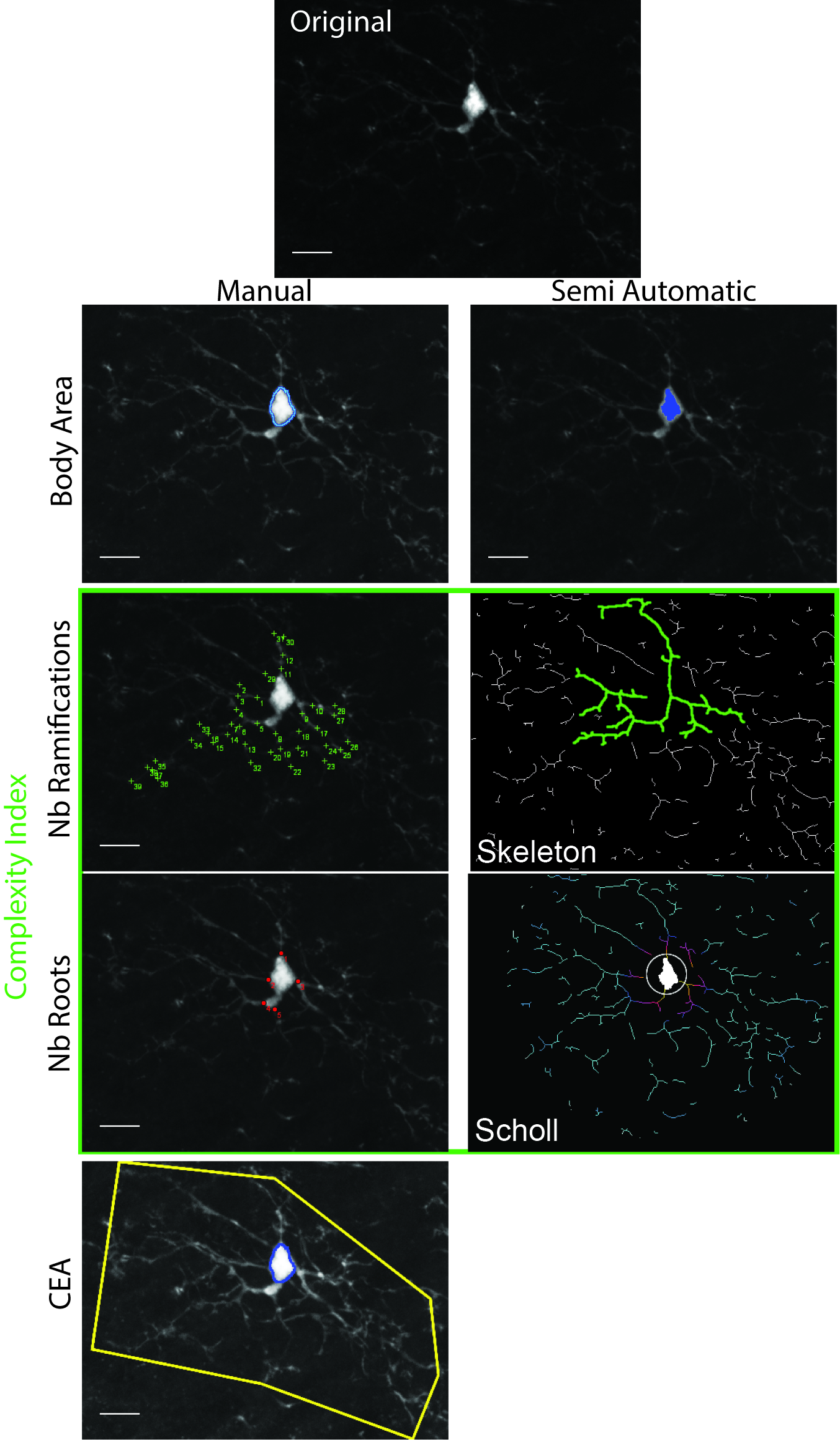

Supplement: Additional file 3: — Data analysis workflow with manual and semi-automated methods. The manual method has been performed using Fiji environment and selection drawing tools to measure the cell body area, the number of roots, ramifications and the CEA. The semi-automated method has been implemented in a Fiji macro using successively Analyze Particles, Skeletonize (2D/3D) and Sholl Analysis plugins. The scale bars equal 10 μm. (TIF 1987 kb) [file 12974_2016_614_MOESM3_ESM.tif]

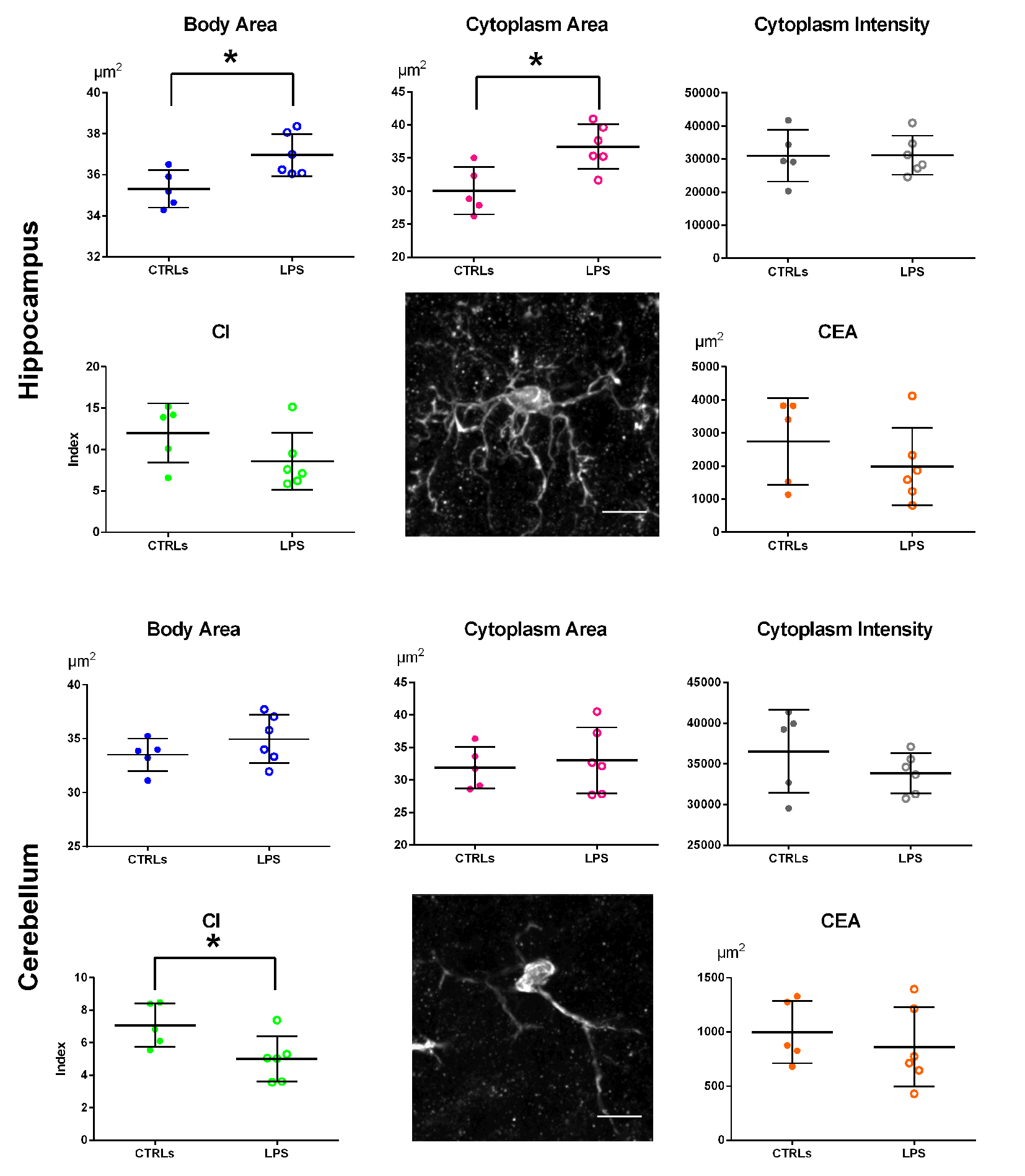

Supplement: Additional file 5: — Characterization of microglial cells by morphological criteria based on Iba-1 expression. Two regions have been explored: hippocampus (H) and cerebellum (C) in the control or LPS conditions. The scatter plots illustrate, by analysed cell, the body area (in blue), cytoplasm area (in pink) and intensity (in grey), CI (in green) or CEA (in orange) characteristics for each animal in both groups. Data shown are means ± SD. The scale bars equal 10 μm. The Mann-Whitney test was used to compare the control and LPS groups (respectively, n = 5 and n = 6).*p < 0.05. (TIF 262 kb) [file 12974_2016_614_MOESM5_ESM.tif]

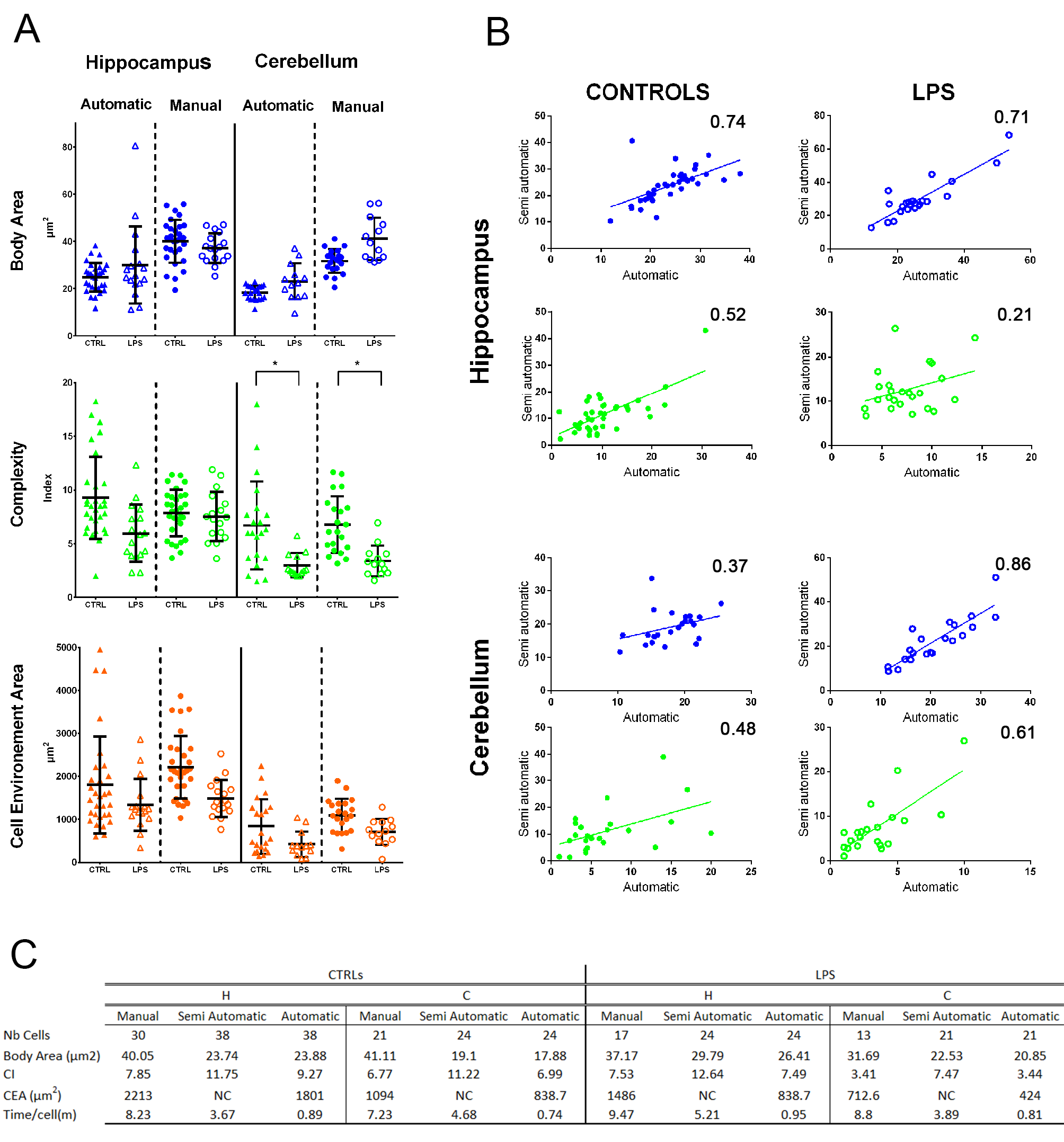

Supplement: Additional file 7: — The comparison of different quantitative analysis methods for microglial morphological criteria by cell. (A) The scatter plots illustrate, at a single cell resolution, the body area, CI or CEA characteristics for the automatic or manual method in the control and LPS groups. The Mann-Whitney test was used to compare the control and LPS groups (respectively, n = 5 and n = 6).*p < 0.05. (B) The correlation plots of two morphological criteria between semi-automatic and automatic analysis with cell body area (in blue) and CI (in green) at a single cell resolution. Values indicate the Spearman correlation coefficient. The lines represent the linear regression. (C) The table indicates the number of cells (Nb cells), the cell body area, the CI, the CEA and the mean analysis time per cell for each condition (control and LPS groups) and each region (hippocampus and cerebellum). (TIF 424 kb) [file 12974_2016_614_MOESM7_ESM.tif]

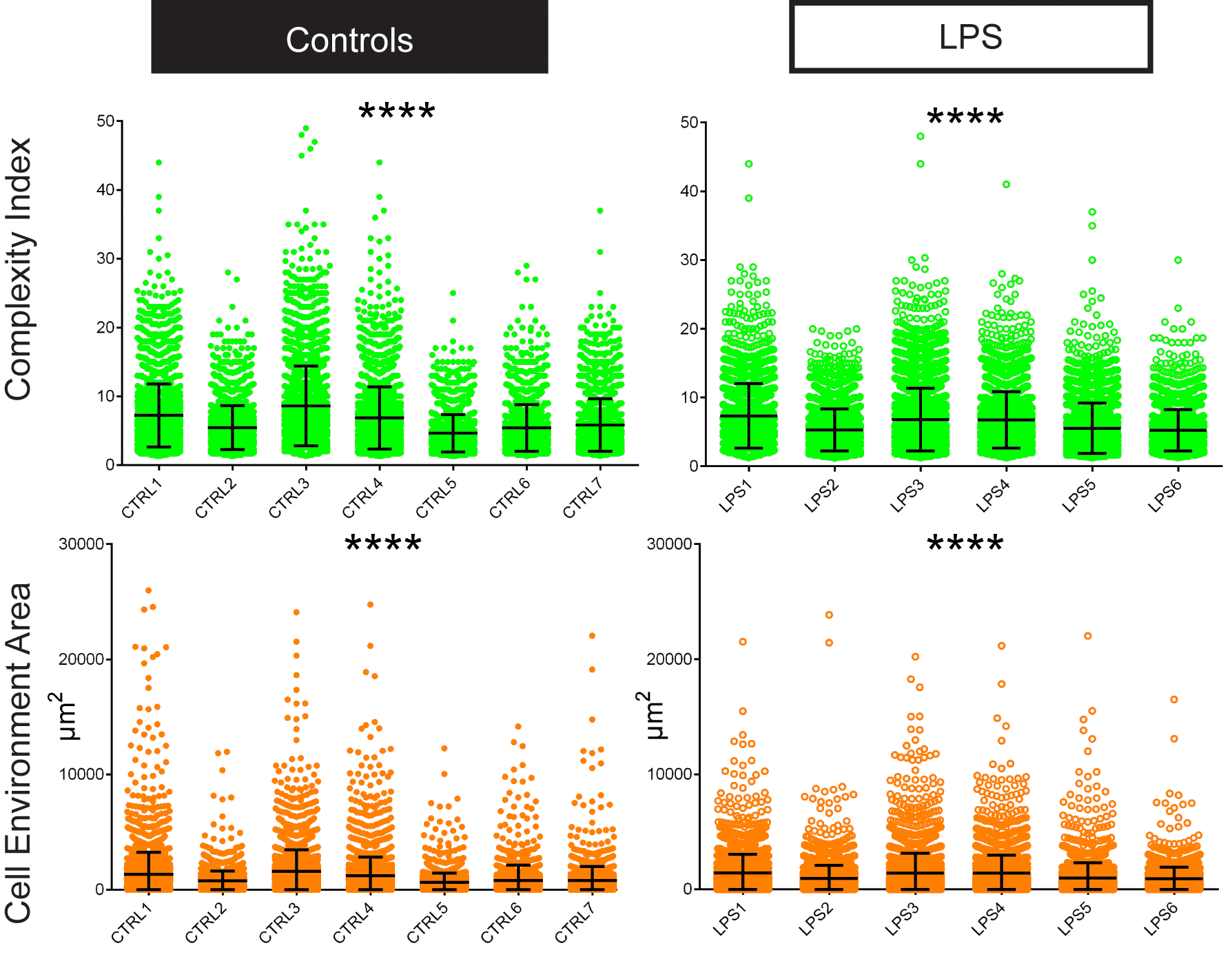

Supplement: Additional file 8: — Quantitative analysis of microglial cell morphology in the whole brain considering CI and CEA. The scatter plots illustrate, at a single cell resolution, the CI or CEA characteristics for each animal in both groups. ANOVA Kruskal-Wallis was used to test the inter-sample heterogeneity. Data shown are means ± SD. ****p < 0.0001. (TIF 438 kb) [file 12974_2016_614_MOESM8_ESM.tif]
