# Supplementary material for: Phenotypic clustering: a novel method for microglial morphology analysis
Source: J Neuroinflammation. 2016 Jun 17;13:153. doi: 10.1186/s12974-016-0614-7 (PMC4912769; doi:10.1186/s12974-016-0614-7)
Supplement: Additional file 4: — Descriptive statistics for microglial cells by condition. (PDF 13 kb) [file 12974_2016_614_MOESM4_ESM.pdf]

Additional file 4. **Descriptive statistics for microglia cells by condition**

| | Controls (n=7) | | | | LPS (n=6) | | | |
|---|---|---|---|---|---|---|---|---|
| **Criteria** | H | FC | S | C | H | FC | S | C |
| **GFP Intensity** | 9985 (1097) | 11307 (1815) | 9877 (1289) | 10419 (1292) | 11360 (2737) | 12109 (1524) | 10508 (1797) | 11869 (2709) |
| **Cell Body Area ($\mu m^2$)** | 22.72 (2.24) | 21.93 (1.98) | 22.87 (2.1) | 20.45 (0.84) | 26.47 (1.33) | 25.34 (1.35) | 26.47 (1.04) | 24.69 (1.98) |
| **Cytoplasm Area ($\mu m^2$)** | 74.42 (8.80) | 63.39 (9.79) | 66.28 (4.09) | 123.4 (24.37) | 149.4 (23.23) | 123.0 (11.39) | 129.2 (14.44) | 177.0 (29.81) |
| **Complexity Index** | 5.89 (1.45) | 5.68 (1.58) | 7.98 (1.80) | 4.49 (0.22) | 4.98 (0.51) | 6.09 (0.81) | 7.30 (1.35) | 3.59 (0.23) |
| **CEA ($\mu m^2$)** | 953.4 (330.7) | 950.6 (400.9) | 1378 (489) | 450 (109.7) | 907.6 (146.2) | 1184 (253.5) | 1499 (396) | 360.1 (145.9) |
| **Density (Cells/$mm^2$)** | 240.7 (53.38) | 370.8 (101.3) | 250.2 (55.59) | 99.12 (23.61) | 174.0 (56.44) | 373.8 (58.42) | 256.5 (55.84) | 62.76 (46.31) |

Values are expressed as mean (± standard deviation of the mean)
