# Supplementary material for: Phenotypic clustering: a novel method for microglial morphology analysis
Source: J Neuroinflammation. 2016 Jun 17;13:153. doi: 10.1186/s12974-016-0614-7 (PMC4912769; doi:10.1186/s12974-016-0614-7)
Supplement: Additional file 6: — Descriptive statistics for amoeboid cells by condition. (PDF 53 kb) [file 12974_2016_614_MOESM6_ESM.pdf]

Additional file 6. **Descriptive statistics for amoeboid cells by condition**

	Controls (n=7)				LPS (n=6)			
**Criteria**	H	FC	S	C	H	FC	S	C
**GFP Intensity**	9062	10334	9075	10120	9666	10332	9400	10744
	(700.8)	(1382)	(991)	(924.5)	(1814)	(1352)	(1565)	(2133)
**Cell Body Area**	19.54	19.96	22.05	18.83	23.94	21.56	22.72	22.96
**(μm^2)**	(1.37)	(1.53)	(1.46)	(1.42)	(2.17)	(1.57)	(3.73)	(2.18)
**Cytoplasm Area**	58.29	56.32	55.74	130.5	102.8	91.43	102.2	230.4
**(μm^2)**	(16.16)	(19.00)	(7.66)	(99.65)	(53.35)	(22.09)	(34.74)	(203.8)
**Density (Cells/mm^2)**	19.12	18.80	11.12	16.65	13.95	11.34	8.46	11.60
	(4.34)	(7.26)	(2.42)	(3.58)	(5.78)	(4.47)	(3.90)	(8.20)
**Frequency (%)**	7.6	5.64	4.41	14.73	7.53	3.13	3.35	17.30
	(2.24)	(2.90)	(1.32)	(3.11)	(2.46)	(1.27)	(1.90)	(5.53)

Values are expressed as mean (± standard deviation of the mean)
